# Harnessing plant growth-promoting bacteria to combat watermelon mosaic virus in squash

**DOI:** 10.1038/s41598-025-92268-2

**Published:** 2025-03-19

**Authors:** Shymaa R. Bashandy, Omima Abdelsater Mohamed, Osama A. Abdalla, A. Elfarash, Mohamed Hemida Abd-Alla

**Affiliations:** 1 Botany and Microbiology Department, Faculty of Science, Assiut University, Assiut, 71516 Egypt; 2 Plant Pathology Department Faculty of Agriculture, Assiut University, Assiut, 71516, Egypt; 3 Genetics Department Faculty of Agriculture, Assiut University, Assiut, 71516, Egypt

**Keywords:** Plant growth-promoting bacteria (PGPB), Sustainable agriculture, Biological control, Crop disease management, Rhizosphere microbiome, Bacterial biostimulants, Microbiology, Environmental microbiology

## Abstract

Plant diseases significantly threaten global food security, with viral infections, particularly Watermelon Mosaic Virus (WMV), causing substantial losses in economically important crops such as squash. This study aims to investigate the efficacy of beneficial bacteria isolated from various plants in promoting growth and mitigating the effects of WMV in squash. Understanding the interactions between plants and beneficial microbes could provide sustainable solutions for managing viral infections in agriculture. Sixty-two bacterial isolates were obtained from the rhizosphere of basil, mint, thyme, and squash plants. Among these, six strains exhibited notable plant growth-promoting activities, including the synthesis of indole acetic acid, solubilization of phosphate and zinc, ammonia production, and activity of 1-aminocyclopropane-1-carboxylate deaminase (ACCD). Morphological observations and 16S rRNA gene sequencing identified these isolates as *Pseudomonas indica*,* Bacillus paramycoides*,* Bacillus thuringiensis*,* Bacillus mycoides*,* Paenibacillus glucanolyticus*, and *Niallia circulans*. In pot experiments, squash plants inoculated with these bacterial strains demonstrated significant reductions in disease severity after being infected with WMV. Specifically, foliar applications of the bacteria resulted in the following reductions in disease severity: *B. mycoides* (87%), *B. thuringiensis* (73%), *Paenibacillus glucanolyticus* (73%), *Niallia circulans* (70%), *B. paramycoides* (65%), and *Pseudomonas indica* (65%). Additionally, plants treated with *B. mycoides* showed increased plant height and shoot dry weight, indicating enhanced growth performance relative to infected controls. Statistical analysis revealed that these growth promotions and disease severity reduction were significant (*p* < 0.05). GC–MS analysis of the six bacterial strains revealed a diverse array of 73 chemical metabolites, including common compounds such as 9-Octadecenoic acid (Z), benzene derivatives, and cyclopentanones. These findings suggest shared metabolic pathways among the strains and indicate potential roles in ecological interactions, plant defense mechanisms, and antiviral properties. These metabolites likely contribute to the observed reductions in viral severity and enhance plant resilience. The study indicates that inoculating squash plants with specific beneficial bacteria, especially *B. mycoides*, through foliar or soil application can significantly decrease the severity of WMV and promote plant growth. This approach offers an environmentally friendly alternative to chemical antiviral treatments and may reduce reliance on pesticides. This research highlights the potential of using plant growth-promoting bacteria (PGPB)as a sustainable approach to control viral infections in crops. Further field trials are necessary to PGPB validate the scalability of these findings and assess their effectiveness under diverse agricultural conditions. Incorporating these beneficial microbes into agricultural practices could enhance the resilience of cropping systems, ultimately fostering sustainable agriculture and enhancing food security.

**Supplementary Information:**

The online version contains supplementary material available at 10.1038/s41598-025-92268-2.

## Introduction

According to ‘The State of Food Security and Nutrition in the World 2020’ report, global food production must increase by more than 50% by 2050 to meet the demands of the growing population^1,2^. This projection highlights the significant challenges in ensuring food security, especially considering factors such as climate change, resource limitations, and the necessity for sustainable agricultural practices. Plant diseases are a major contributor to global crop losses, accounting for 40% of the total loss and causing an economic impact of over USD 220 billion, as reported by the World Health Organization^3,4^. Among biotic stresses, plant viruses are significant culprits, leading to severe epidemics in economically important crops. More than 80% of plant viruses have RNA genomes, and approximately 50% of plant virus species that cause diseases in plants are intracellular parasites^5^. The WMV is a significant plant virus globally, affecting a diverse array of hosts, with over 170 plant species from 27 different families being susceptible^6^. WMV is classified under the genus *Potyvirus* within the Potyviridae family, and it is distinguished by its flexible, filamentous particles that are approximately 750 nm in length^7,8^. WMV is transmitted both mechanically via plant sap and through aphids in a non-persistent manner^9,10^. The impact of WMV on cucurbit plants is severe, leading to a variety of symptoms depending on the virus isolate, host cultivar, and plant age^11^.

Squash (*Cucurbita pepo* L.) is a vital vegetable crop grown in Egypt and other countries, known for its high nutritional content, including vitamins (B, C, and A), minerals (especially K+), flavonoids, and phenolic compounds^12,13^. Despite its nutritional value and low glycemic index, squash remains susceptible to viral diseases, causing economic losses due to foliage and fruit damage^14^. The cultivation area for squash has expanded significantly in recent years, especially in newly reclaimed regions for both open and protected plantations^15^. Managing plant viral diseases is particularly challenging because chemical control measures are generally ineffective against intracellular pathogens^16^. Plant viruses reside within the cells of their host plants, making it difficult for chemical treatments to reach and eliminate them without harming the plant itself^17^. Unlike fungi or bacteria, which can often be targeted on the plant’s surface or in the soil, viruses are protected within the plant’s cellular structure^17^. Most chemical pesticides are designed to target specific biochemical pathways or structures in pathogens. However, viruses lack many of these targets because they rely on the host plant’s cellular machinery for replication, making it challenging to develop chemicals that can specifically target viruses without affecting the plant^18^. Excessive use of chemical treatments can lead to the recombination of viral genomes, potentially creating more aggressive and resistant strains^17^. For instance, the use of certain antiviral chemicals has been shown to induce mutations in viral RNA, leading to the emergence of new, more virulent strains^18^. Additionally, the use of chemical pesticides can have detrimental effects on the environment and human health, as the overuse of pesticides can lead to the accumulation of harmful residues in the soil and water, affecting non-target organisms and potentially entering the food chain. Given these limitations, alternative strategies such as the use of resistant plant varieties, biological control agents, and advanced biotechnological tools like CRISPR/Cas9 are being explored as more sustainable and effective ways to manage plant viral diseases^17^.

Eco-friendly crop disease management is essential for sustainable farming practices. Plant growth-promoting bacteria (PGPB) play a crucial role in controlling plant pathogens by enhancing seed germination, root development, and water uptake. They promote plant growth through hormone production and improved nutrient absorption^19^. In the 21st century, managing crop diseases caused by fungi, viruses, and bacteria is crucial due to their significant impact on crop yields and quality. Plant viral diseases are on the rise due to factors such as climate change, trade, and rapid virus evolution^20^. PGPB are essential in this context as they enhance seed germination, root development, and water uptake^21^. They also promote plant growth by producing hormones and improving nutrient uptake, while rebalancing the microbial ecosystem in favor of beneficial microorganisms, thereby suppressing various diseases. PGPB also shifts the microbial balance in favor of beneficial microorganisms, suppressing various diseases. The use of PGPB is increasing globally, with potential for wider application in sustainable agriculture^22,23^. This study aims to evaluate the effectiveness of bacterial isolates from basil, mint, thyme, and squash in promoting plant growth and managing WMV in cucurbits. Furthermore, the research aims to investigate the potential PGPB strains in inhibiting WMV infection and symptoms, as well as to elucidate the resistance mechanisms by analyzing the bacterial culture supernatant using GC-MS.

## Materials and methods

### Virus isolation, identification, and molecular characterization

**Fig. 1:**
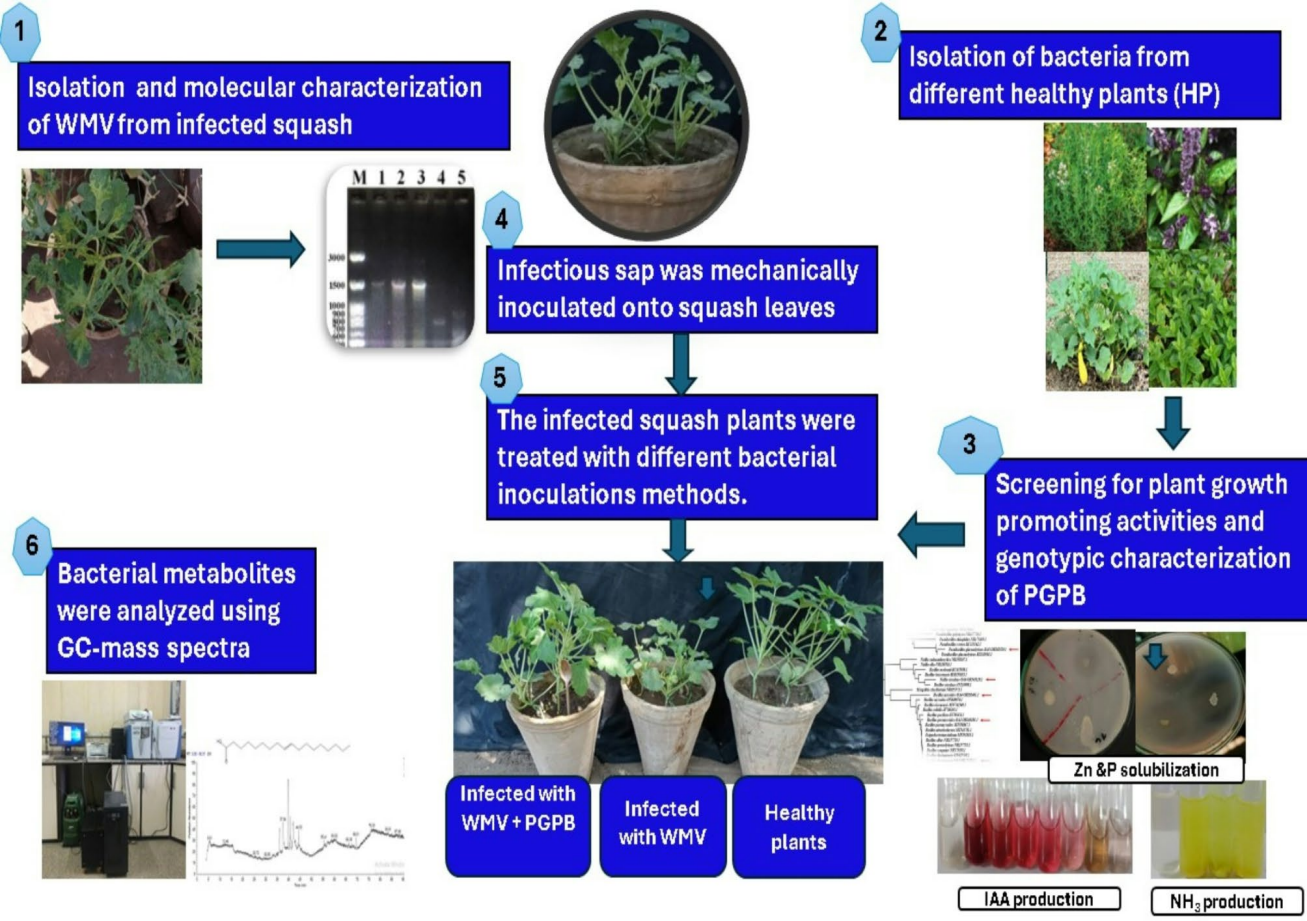
Schematic flowchart of the experimental design used in the study.

The infected young squash leaves showing leaf mosaic and deformations at the farm of the Faculty of Agriculture, Assiut University, Egypt, were ground in a phosphate buffer solution (pH 7.2). The infectious sap was then mechanically inoculated onto squash leaves to induce chlorotic local lesion symptoms^8^. A single local lesion developed at 6 days post-viral inoculation (dpi) and was used as a source for the viral isolate, which was maintained on squash plants through mechanical inoculation. The study’s experimental design was illustrated in a schematic flowchart (Fig. 1).

Total RNA was extracted from squash plants showing symptoms similar to WMV using the ABT Total RNA Mini Extraction kit following the manufacturer’s instructions (Applied Biotechnology). cDNA was synthesized from 1 µg of RNA using the ABT H-minus kit (Applied Biotechnology). RT-PCR was performed in a two-step process. Initially, cDNA was synthesized from 1 µg of RNA using the ABT H-minus kit (Applied Biotechnology) according to the manufacturer’s instructions. Subsequently, PCR was carried out using 2 µl of the resulting cDNA and WMV-CP gene-specific forward and reverse primers: WMV-F CACATTACTTGGAGCTAAAG; WMV-R ATATGCTTCCGCTGCATCTG, as described by^24^. The PCR protocol included an initial denaturation at 94 °C for 2 min, followed by 30 cycles of denaturation at 94 °C for 30 s, annealing at 55 °C for 30 s, and elongation at 72 °C for 40 s, with a final extension step at 72 °C for 10 min. Finally, the PCR products were separated on a 1.5% agarose gel, stained with ethidium bromide, and analyzed using a gel documentation system.

### Bacterial isolation

Rhizoplane and endophytic bacteria were isolated from basil (*Ocimum basilicum*), mint (*Mentha viridis*), thyme (*Thymus vulgaris*), and squash (*Cucurbita pepo*) plants grown in the botanical garden of the Botany and Microbiology Department at the Faculty of Science, Assiut University. Permission was obtained from Assiut University authorities to collect these plants, a WMV isolate, and bacterial isolates from the university’s botanical garden. For rhizoplane bacteria, roots were cut into 1 cm² segments, washed with 5% sodium hypochlorite (NaOCl) for 3 min, and then rinsed with sterilized distilled water. The segments were dried and placed on agar medium for incubation at 28 ± 1 °C for 7 days and at 30 ± 1 °C for 48 h. The isolated bacteria were identified and quantified as colony-forming units (CFU) per 15 root segments. For endophytic bacteria, roots were washed, treated with alcohol, NaOCl, and sterilized distilled water, dried, and plated on agar medium for incubation. The developing bacteria were identified and quantified as CFU per 15 root segments. PGPB were isolated using Nutrient Agar (NA) medium^25^ and Ashby’s nitrogen-free mannitol medium, which consists of mannitol (20 g/L), agar (15 g/L), calcium carbonate (5 g/L), sodium chloride (0.2 g/L), magnesium sulfate (0.2 g/L), dipotassium phosphate (0.2 g/L), and potassium sulfate (0.1 g/L)^26^.

### Screening for plant growth-promoting activities

#### Phosphate and zinc solubilization

Phosphate and zinc solubilization tests are crucial for plant growth promotion because they help identify microorganisms that can convert insoluble forms of these essential nutrients into forms that plants can readily absorb. Phosphorus is vital for various plant processes, including photosynthesis, energy transfer, and nutrient transport. However, much of the phosphorus in soil is in an insoluble form that plants cannot use^27^. Phosphate-solubilizing microorganisms release organic acids and enzymes that convert these insoluble phosphates into soluble forms, enhancing plant growth and yield^28,29^. Similarly, zinc is an essential micronutrient involved in enzyme activation, protein synthesis, and growth regulation. Zinc-solubilizing bacteria can convert insoluble zinc compounds into soluble forms, making them available to plants. This process not only improves plant health and productivity but also reduces the need for chemical fertilizers, promoting sustainable agriculture^30,31^.

To assess phosphate solubilization, the isolates were cultured on Pikovskaya medium (PVK) containing 10 g/L glucose; 5 g/L Ca_3_(PO_4_)_2_; 0.5 g/L (NH_4_)_2_SO_4_; 0.2 g/L NaCl; 0.1 g/L MgSO_4_·7 H_2_O; 0.2 g/L KCl; 0.5 g/L yeast extract; 0.5 g/L MnSO_4_·H_2_O; and 0.002 g/L FeSO_4_·7 H_2_O)^32^. After 5 days of incubation at 30 °C, clear haloes formed around the colonies, indicating phosphate solubilization activity. For zinc solubilization, bacterial isolates were cultured on mineral salt medium with insoluble ZnO and incubated at 28 °C. After 5 days of incubation at 28 °C, clear haloes formed around the colonies, indicating phosphate and zinc solubilization activity. The diameters of the colonies and halo zones were measured under standard laboratory conditions using a ruler to ensure accuracy. The phosphate and zinc solubilization indices were calculated using the formula: (size of colony + size of clear zone) (cm)/diameter of colony (cm)^33,34^.

### Indole acetic acid (IAA) production

To quantify the production of indole-3-acetic acid (IAA) by each isolate, the isolates were cultured in nutrient broth and Ashby’s nitrogen-free mannitol media supplemented with 0.2 g/l L-tryptophan. Incubation was performed at 28 ± 2 °C on a rotary shaker (150 rpm) for 2 days with experiments performed in triplicate. Following incubation, the broth was centrifuged, and 1 mL of the supernatant was mixed with 2 mL of Salkowski’s reagent (2.02 gm FeCl_3_ + 500 mL distilled water and 300 mL conc. H_2_SO_4_) and kept in the dark^35^. Absorbance at 540 nm was measured using Evolution 300 UV-Visible spectrophotometer (Thermo Fischer Scientific, USA)^36,37^. A calibration curve was generated using pure IAA, and the IAA concentration was expressed as µg/mg cell protein.

### Ammonia (NH_3_) production

Ammonia estimation was conducted by growing each bacterial isolate in 10 mL of peptone water broth at 30 °C for 48–72 h. After centrifugation, 0.5 mL of Nessler’s reagent was added to each culture tube and allowed to react for 15 min at room temperature. The development of a brown to yellow color indicated the presence of ammonia^38^.

### 1-aminocyclopropane-1-carboxylate deaminase (ACC) activity

The modified method was utilized to determine ACC-deaminase activity of bacterial strains^39^. The assessment of bacterial ACC deaminase activity and root-growth enhancement both necessitate bacterial growth conditions conducive to ACC deaminase induction. Initially, bacteria are cultured in rich medium and then transferred to minimal medium with ACC as the sole nitrogen source. Cultures are incubated overnight in a shaking water bath at 200 rpm at the optimal temperature for the bacterial strain. The biomass is harvested by centrifugation at 8000 g for 10 min at 4 °C. A 0.5 M ACC solution is added to the cell suspension, and a 45 µL aliquot is taken to achieve a final ACC concentration of 3.0 mM. The bacterial cells are returned to the shaking water bath to induce ACC deaminase activity at 200 rpm for 24 h at the same temperature as the overnight incubation, either 25–30 °C. Subsequently, the cells are assayed for ACC deaminase activity following the method outlined by^40^. The values obtained from this protocol are used to estimate the amount of µmol of α-ketobutyrate produced by the tested strains. These values are compared to a standard curve generated by adding 2 mL of 2,4-dinitrophenylhydrazine to each standard within a range of 0.1 to 1 µmol of α-ketobutyrate. The solution is vortexed and incubated at 30 °C for 30 min, and the absorbance is measured at 540 nm using Evolution 300 UV-Visible spectrophotometer (Thermo Fischer Scientific, USA) after the addition of 2 mL of 2 mol/L NaOH.

### Phenotypic and genotypic characterization of bacterial isolates

The bacterial isolates exhibiting the highest plant growth-promoting activities were characterized both phenotypically and genotypically. Phenotypic characterization followed the methods outlined in Bergey’s Manual^41^. For genotypic characterization, the isolates were identified by 16S rRNA gene sequencing at SolGent Company (Solgent Co., Ltd, Bio Industry Development Site, 63 − 10 Hwaan-Dong, Yuseong-Gu, Daejeon, South Korea). The obtained sequences were analyzed using the Basic Local Alignment Search Tool (BLAST) from the National Center for Biotechnology Information (NCBI) website. Sequence reads were edited and assembled using BioEdit version 7.0.4 (http://www.mbio.ncsu.edu/BioEdit/bioedit.html). BLASTN searches were performed using the NCBI server (http://www.ncbi.nlm.nih.gov/blast/Blast.cgi). Phylogenetic trees derived from 16S rRNA gene sequences were constructed using Mega11 incorporating 16S rRNA gene sequences of various standard bacterial strains obtained from GenBank. The nucleotide sequence of the bacterial isolate has been deposited in the GenBank database with the accession number.

### Plant culture and experimental conditions

A pot experiment was conducted in the greenhouse of the Faculty of Agriculture at Assiut University, Assiut, Egypt, using a randomized complete block design. Each pot contained 3 kg of sterilized soil with a 3:1 clay-to-sand ratio (by weight), and six squash seeds were planted per pot. The soil underwent thorough sterilization by autoclaving at 121 °C and 1.5 bar pressure for 30 min in three separate cycles, with a 60-minute cooling interval between each cycle. The experiment took place under natural photoperiod conditions at approximately 27°18′ N latitude during the late summer season over three consecutive years. Seeds were sown in the first week of May each year. During the growing months (May to July), maximum temperatures typically exceeded 35 °C, with average daytime temperatures above 30 °C. Plants were irrigated as needed.

Squash plants were subjected to various bacterial inoculation treatments (10^7^ CFU/mL) to assess their effects. The treatments included soil inoculation, where a bacterial suspension was prepared by culturing the isolates on nutrient broth (NB) and incubating at 28 °C for 48 h. The concentration was adjusted to 10^7^ CFU/mL, and 100 mL of the bacterial suspension was applied to the soil in each pot at planting, with inoculation repeated every two weeks. Seed soaking involved soaking squash seeds in 50 mL of each PGPB suspension (10^7^CFU/mL) for 4 h before planting. Foliar inoculation was performed 10 days after germination, with 50 mL of each bacterial suspension sprayed onto the cotyledon leaves of each pot using a sprinkler and repeated every two weeks. Control treatments included healthy control plants (without WMV and without bacterial inoculations) and infected control plants (infected with WMV, without bacterial inoculation). Twelve days post-planting, the plants were inoculated with WMV. The leaves from the infected plants were ground using a mortar and pestle with potassium phosphate buffer (pH 7.2), and the resulting homogenate was filtered through cheesecloth. The leaves of the test plants were dusted with 400 mesh carborundum before being mechanically inoculated with the sap. After inoculation, the plants were rinsed with distilled water, kept in a greenhouse, and monitored for symptom development over four to six weeks. Each treatment was replicated three times. The pots were watered with tap water to maintain field capacity, and seven days after sprouting, the seedlings were thinned to three per pot. Disease incidence was determined as a percentage of diseased plants, and disease severity was assessed using a four-class scale: 0 = no symptoms, 1 = yellowing in lower leaves, 2 = yellowing and mosaic, 3 = severe mosaic and mottling, and 4 = malformed leaves, stunted growth, severe mosaic, or plant death. Disease severity values were calculated using the formula described by^42^.

$${\text{Disease}}\;{\text{Severity}} = \frac{{\sum {\left( {{\text{Disease}}\;{\text{grade}}\, \times \,{\text{Number}}\;{\text{of}}\;{\text{plants}}\;{\text{in}}\;{\text{each}}\;{\text{grade}}} \right)} }}{{({\text{Total}}\;{\text{number}}\;{\text{of}}\;{\text{plants}}) \times ({\text{The}}\;{\text{highest}}\;{\text{disease}}\;{\text{grade}})}}$$

The plants were harvested six weeks after sowing when squash plants are typically in the vegetative growth stage, characterized by rapid development of leaves, stems, and possibly the early stages of flowering. Plant height, root length, shoot dry matter content, and root dry matter content were measured. The plant material was dried at 80 °C for 48 h to determine the dry matter content.

### Physiological and biochemical analyses

#### Evaluation of total chlorophyll and carotenoids

The chlorophyll a, chlorophyll b and carotenoid contents in leaves were evaluated following the protocol described by^43^. Fresh leaves were immersed in 95% ethyl alcohol overnight, and absorbances were measured at 663, 644, and 452 nm wavelengths using Evolution 300 UV-Visible spectrophotometer (Thermo Fischer Scientific, USA).

#### Evaluation of total soluble carbohydrates

Total soluble carbohydrates (mg/g dry weight) were determined using a standard glucose curve. The soluble carbohydrate content in the squash plant groups was measured using the phenol method^44^. Initially, 0.5 g of fresh squash leaves were mixed with 5 mL of distilled water and 0.5 mL of 5% phenol, followed by the addition of 2.5 mL of concentrated H_2_SO_4_. After centrifugation at 8000 g for 10 min, the absorbance of the resulting yellow-orange color was measured at 490 nm using Evolution 300 UV-Visible spectrophotometer (Thermo Fischer Scientific, USA). A calibration curve was constructed using D-glucose, and the concentration of soluble sugars was determined as mg g^− 1^ DW. The total soluble carbohydrate content (mg/g dry weight) was calculated using the standard glucose curve.

#### Identification of bacterial bioactive compounds through gas chromatography–mass spectrometry (GC–MS)

The Six bacterial isolates were cultured in 1 L of Nutrient Broth medium for 2 days. Following cultivation, the culture was centrifuged at 10,000 g for 10 min, and the supernatant was transferred to conical flasks. In each flask, ethyl acetate was added to the supernatant in a 1:1 ratio, followed by shaking and allowing it to stand overnight to ensure comprehensive extraction. The resulting extraction solution was then concentrated and dried using a rotary evaporator. Each extract was weighed and dissolved in methanol in preparation for analysis using a Thermo Scientific ISQ7000 single quadrupole GC-MS system, coupled with a mass detector in split mode (Chemistry Department, Faculty of Science, Assiut University). Helium gas served as the carrier gas at a constant flow rate of 1 mL/min. The GC-MS oven temperature was programmed to start at 100 °C, gradually increasing to 280 °C over a period of 20 min. The mass spectrometer scanned at intervals of 0.2 s, covering a mass range of 40–750 amu. The identification of chemical components was achieved by comparing the retention times of the analytes with those of internal standards treated under the same experimental conditions, and the results were analyzed using the National Institute of Standards and Technology spectral library version 11.

Data analysis was performed using the National Institute of Standards and Technology (NIST) spectral library version 11, which facilitated the precise identification and quantification of the compounds present in the extracts^45^.

### Statistical analysis

Statistical significance was assessed using GraphPad Prism software with analysis of variance and a significance threshold of *p* ≤ 0.05. Data analysis was also conducted using IBM SPSS Statistics Version 25. The results are presented as means and standard errors calculated from three replicates. Statistical significance is indicated by letters in descending order (a > b > c), with identical letters denoting equal significance.

## Results

### Virus isolation and identification

**Fig. 2:**
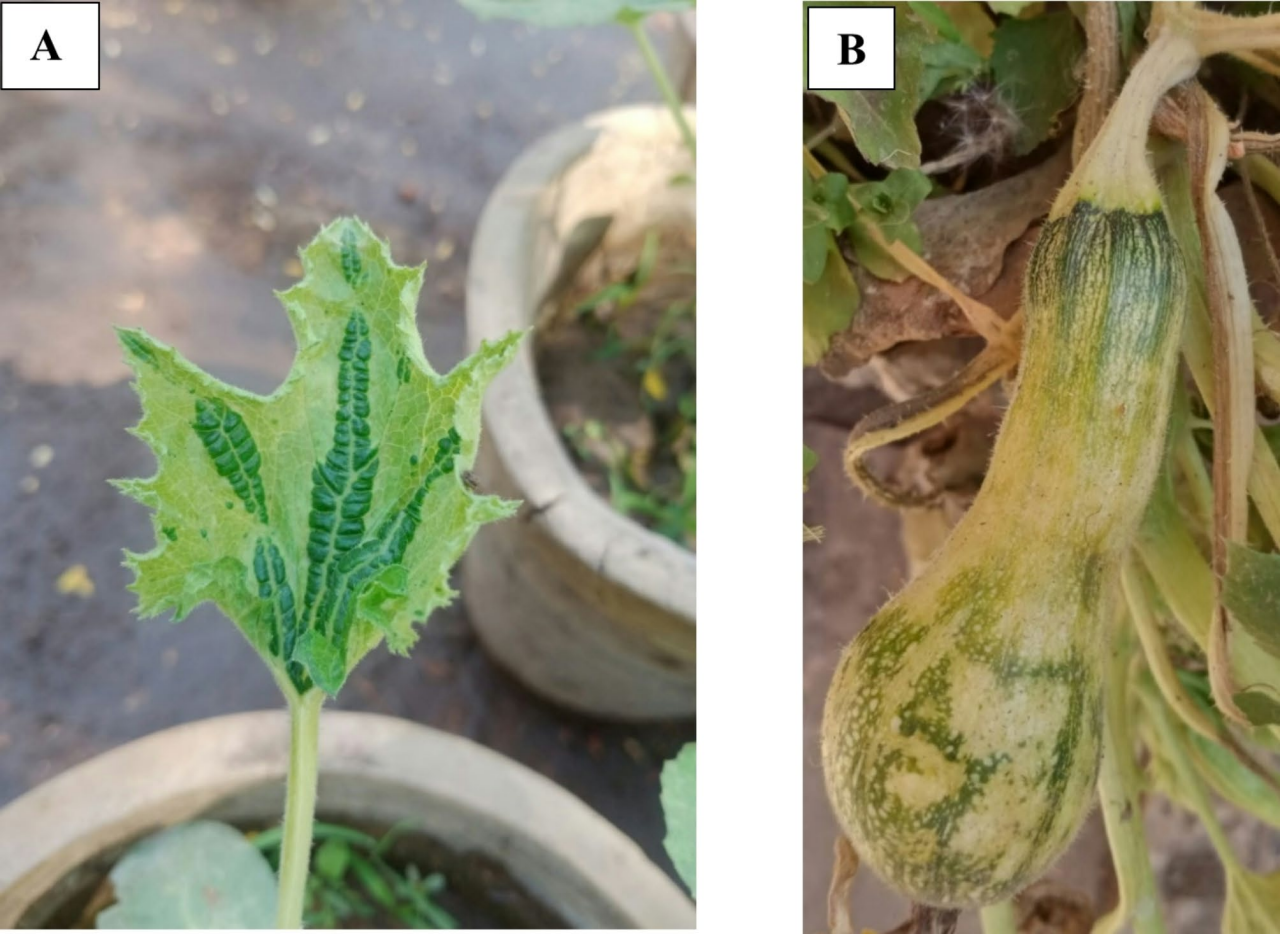
WMV-infected squash (*Cucirbita pepo* L.) plant exhibiting mottling (**A**), mosaic and fruit deformation symptoms (**B**).

Squash plants infected with a virus exhibited symptoms such as green mosaic, blistering, vein banding, and malformation. The virus was isolated and transferred to squash plants (*Cucurbita pepo*) via single lesion transfers. The virus was then propagated in *C. pepo* to establish a pure virus source. Infected plants showed symptoms ranging from mild to severe mosaic, leaf distortion, and fruit deformation (Fig. 2A, B). The isolated virus was identified as WMV using RT-PCR, with a DNA fragment of approximately 1500 bp confirming its identity (Supplementary Figure S1).

### Bacterial isolation and screening for plant growth promoting activities

In the current experiment, we performed a 48-hour evaluation of the plant growth-promoting capabilities of 62 different bacterial isolates. Our analysis focused on their ability to produce IAA, ACC deaminase, and ammonia, as well as their capacity to solubilize phosphate and zinc. Notably, bacterial isolates OA2, OA7, OA8, OA15, OA52, and OA56 exhibited significant plant growth-promoting activity, as shown in Table 1. Among these isolates, OA15 produced 34.081 µg/mL of IAA, while OA8 and OA2 had higher levels of 122.081 µg/mL and 184.526 µg/mL, respectively.

**Table 1:** Screening for plant growth promoting activities producing by bacterial isolates*.

Bacterial isolates	IAA production (µg/mL)	ACC production (nmol α-ketobutyrate mg protein^− 1^ h^− 1^)	Ammonia production	Phosphate solubilizing index (cm)	Zinc solubilizing index (cm)
*P. indica* (OA7)	27.015^c^	1457.2^ab^	-	4.43^b^	4.92^b^
*B. paramycoides* (OA15)	34.081^c^	1235.8^ab^	-	6^a^	2.58^c^
*B. thuringiensis *(OA52	7.281^d^	0^b^	-	6.09^a^	7^a^
*B. mycoides *(OA2)	184.526^a^	1870.1^ab^	-	0^c^	0^d^
*Paenibacillus glucanolyticus* (OA8)	122.081^b^	2554.6^a^	-	0^c^	0^d^
*Niallia circulans* (OA56)	2.170^d^	1770.7^ab^	++	3.14^b^	4.71^b^

*Means with same letter in each column are not significantly different at the 0.05 level using Duncan LSD test.

Moreover, OA7 and OA56 exhibited similar ACC production levels, ranging from 1457.12 to 1770.7 (nmol α-ketobutyrate mg protein^− 1^ h^− 1^). In contrast, OA2 and OA8 showed high ACC deaminase production, with values of 1870.121 and 2554.599 (nmol α-ketobutyrate mg protein^− 1^ h^− 1^), respectively. Furthermore, OA7 and OA56 display moderate phosphate solubilizing indices, with values of 4.43 cm and 3.14 cm, respectively (Table 1). Lastly, OA15 and OA52 demonstrated high phosphate solubilizing indices, with values of 6 cm and 6.09 cm, respectively.

### Phenotypic and genotypic characterization of bacterial isolates

The bacterial isolates were identified based on their phenotypic traits. For instance, bacterial isolate OA2 is Gram-positive, non-motile, and forms expansive hairy colonies with characteristic swirls. In contrast, bacterial isolate OA7 is Gram-negative, non-spore-forming, and rod-shaped, with motility. Bacterial isolate OA8 is rod-shaped, Gram-positive, with flat, white opaque colonies. Bacterial isolate OA15 is a non-motile, rod-shaped bacterium, while bacterial isolate OA52 is Gram-positive and spore-forming. Lastly, OA56 is an aerobic, Gram-stain, catalase, and oxidase-positive, spore-forming, motile rod. The detailed summary of the biochemical and physiological characteristics of these isolates is presented in Table 2.

**Table 2:** Biochemical tests of bacterial isolate OA2, OA7, OA8, OA15, OA52, OA56 exhibited the highest plant growth-promoting activities.

Test	OA7	OA15	OA52	OA2	OA8	OA56
Starch hydrolysis	++	++	++	+++	-	+
Gelatin hydrolysis	+	++	+++	++	-	+
Lipase test	-	+	-	+	-	-
Catalase test	+	++	++	-	-	+
Urease test	-	-	-	++	-	-
H_2_S	+	+	+	-	+	-
Carbohydrates test						
Glucose	++	++	++	++	++	++
Sucrose	+	++	++	++	+	++
Lactose	++	+	-	++	++	+++
Fructose	+	++	++	++	+	+++
Maltose	+	++	++	++	+	++
Mannitol	+	-	-	++	+	++
Glycerol	+	+	+	++	++	++
Sorbitol	+	-	-	++	+	+++

**Fig. 3:**
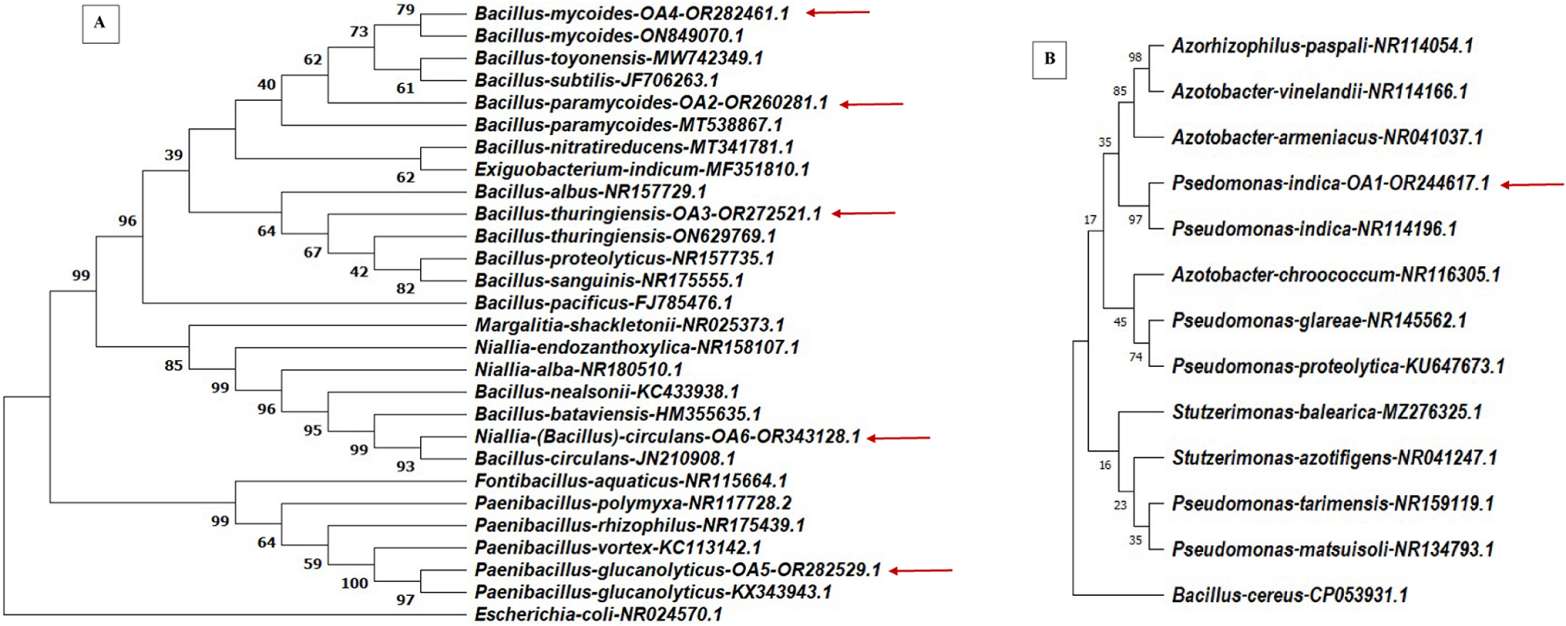
A phylogenetic tree based on neighbour-joining method using MEGA11. The percentage of replicate trees in which the associated taxa clustered together in the bootstrap test (1000 replicates) are shown next to the branches. Red arrows indicate the positions of selected plant growth-promoting bacterial strains (OR244617.1, OR260281.1, OR272521.1, OR282461.1, OR282529.1, OR343128.1). *Escherichia coli* (NR024570.1) (**A**) and *Bacillus cereus* (KU377337.1) (**B**) were used as an out of group in the phylogenetic trees. Numbers are the accession number of the sequence in the database (GenBank).

Molecular identification by 16S rRNA gene sequencing revealed that the almost complete 1421-bp 16S rRNA gene sequence of OA2 shares 96.9% identity with *Bacillus mycoides* (ON849070.1). Similarly, the nearly complete 1425-bp 16S rRNA gene sequence of OA8 shows 98.80% identity with *Paenibacillus glucanolyticus* strain PD4 (KX343943.1). Furthermore, the nearly complete 1444-bp 16S rRNA gene sequence of OA15 exhibits 99.58% identity with *Bacillus paramycoides* strain 8929 (MT538867.1). Additionally, the genetic analysis showed that the nearly complete 1422-bp 16S rRNA gene sequence of OA52 matches 100% with *Bacillus thuringiensis* (ON629769.1), while the nearly complete 768-bp 16S rRNA gene sequence of OA56 displays 98.05% identity with *Bacillus circulans* (JN210908). A phylogenetic tree of 16S rRNA gene sequences was plotted (Fig. 3A), and the annotated sequences were deposited in GenBank under the accession numbers OR282461.1, OR282529.1, OR260281.1, OR272521.1, and OR343128.1, respectively.

Genotypic analysis showed that the nearly complete 1398-bp 16S rRNA gene sequence of OA7 had 99.93% identity with *Pseudomonas indica* strain NBRC 103,045 (NR114196). A phylogenetic tree of 16S rRNA gene sequences was plotted (Fig. 3B), and OA7 was selected based on its strong plant growth promotion ability. The annotated sequence was deposited in GenBank under the accession number OR244617.1.

### Nucleotide sequence accession number

The nucleotide sequences of bacterial isolates (OA2, OA7, OA8, OA15, OA52, OA56) isolated from basil (*Ocimum basilicum*), mint (*Mentha viridis*), thyme (*Thymus vulgaris*), and squash (*Cucurbita pepo* L.) plants were deposited in the GenBank nucleotide sequence database under the accession numbers OR282461.1, OR244617.1, OR282529.1, OR260281.1, OR272521.1, and OR343128.1, respectively.

### Effect of inoculation of PGPB strains on squash plant growth infected with WMV

In a pot experiment, various PGPB (*Pseudomonas indica* OR244617, *Bacillus paramycoides* OR260281, *Bacillus thuringiensis* OR272521, *Bacillus mycoides* OR282461, *Paenibacillus glucanolyticus* OR282529, and *Niallia circulans* OR343128) were applied separately to squash plants using three different methods: seed soaking, soil inoculation, and foliar inoculation. The treated plants were then exposed to WMV infection and compared to control plants that were not inoculated with bacteria.

### Plant height

**Fig. 4:**
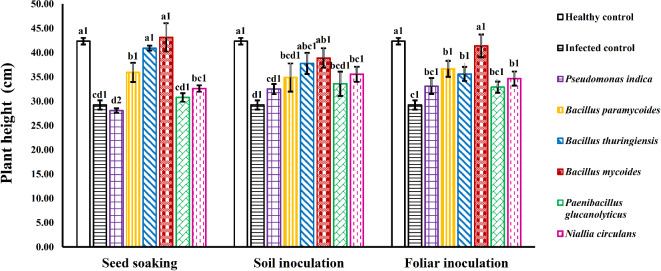
Influence of various bacterial strains and treatment methods on the height of squash plants infected with WMV. Each column represents the mean value of three replicates ± SE (vertical bars). Means with the same letters and with the same numbers among treatment methods are not significantly different at the 0.05 level using Duncan LSD test.

After six weeks of growth and treatment, squash plants infected with WMV and treated with different bacterial strains showed a noticeable increase in height compared to infected controls (plants exposed to WMV without bacterial treatment (Fig. 4A&B). All treatments of infected squash plants with WMV using *Bacillus mycoides* or *Bacillus thuringiensis*, whether applied through seed soaking or foliar inoculation, resulted in a significant increase compared to infected controls. Importantly, there was no significant difference between these treated plants and those that were uninfected (without viral infection or bacterial inoculation) (Fig. 4A&B).

### Pigment contents

**Fig. 5:**
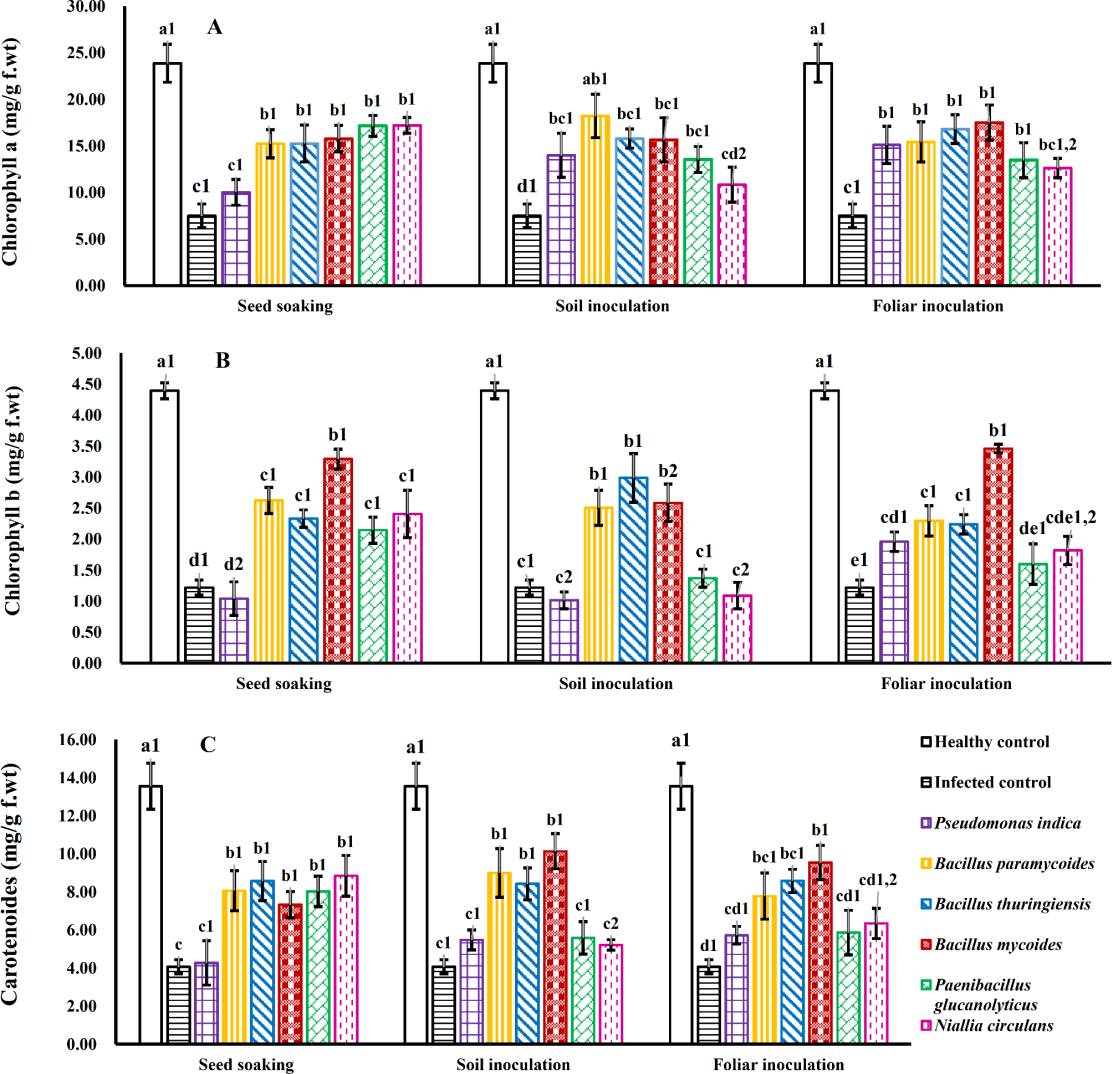
Influence of various bacterial strains and treatment methods on pigment contents of squash plants infected with WMV. Each column represents the mean value of three replicates ± SE (vertical bars). Means with the same letters among bacterial isolates and with the same numbers among treatment methods are not significantly different at the 0.05 level using Duncan LSD test.

Figure 5 illustrates that WMV infection caused a marked decrease in chlorophyll and carotenoid levels in leaf tissues. In contrast, the application of various strains of PGPB significantly enhanced chlorophyll a, chlorophyll b (Fig. 5A & B) and carotenoid contents (Fig. 5C) in WMV-infected squash plants compared to those that were solely infected.

### Carbohydrate content

**Fig. 6:**
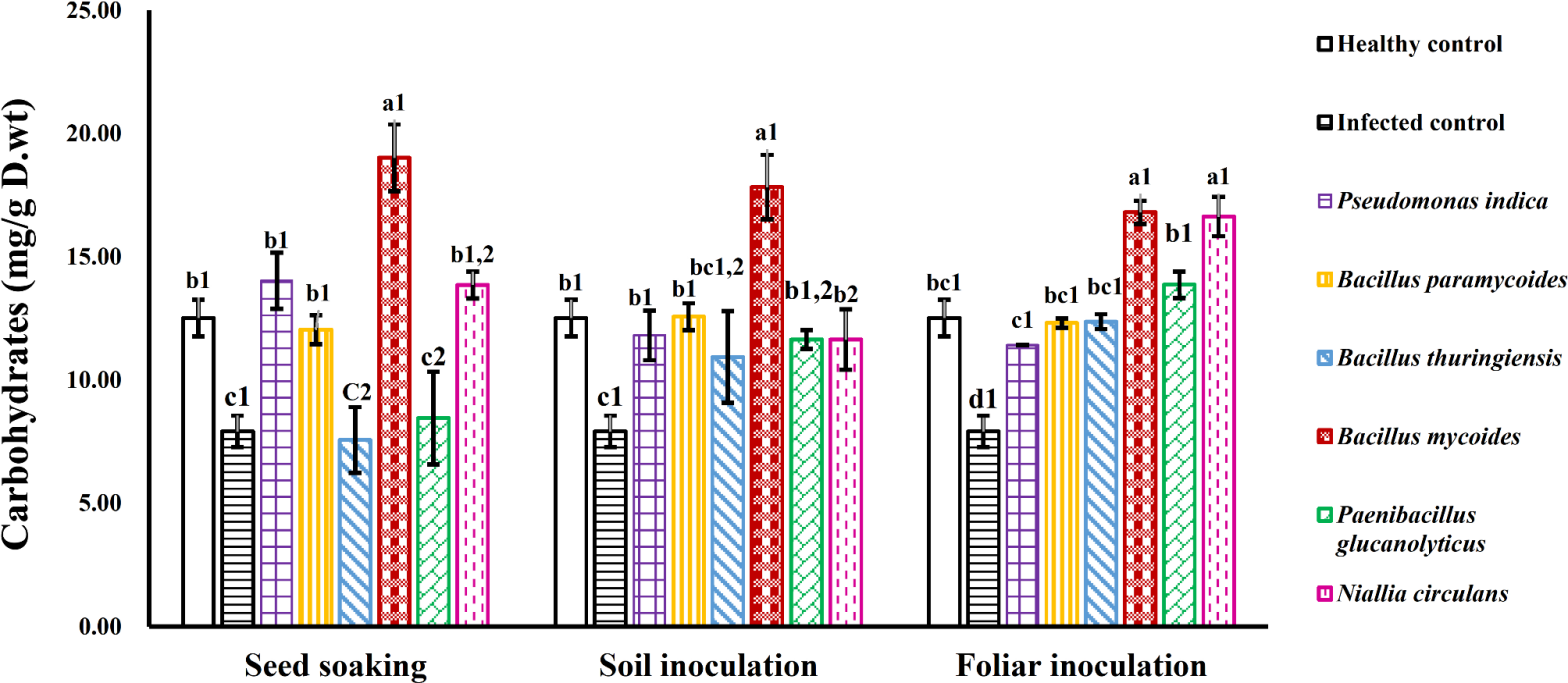
Influence of various bacterial strains and treatment methods on the carbohydrate contents of squash plants infected with WMV. Each column represents the mean value of three replicates ± SE (vertical bars). Means with the same letters among bacterial isolates and with the same numbers among treatment methods are not significantly different at the 0.05 level using Duncan LSD test.

WMV-infected plants (without bacterial inoculation) showed a significant reduction in total soluble carbohydrates to 7.9 ± 0.02 mg/g DW, representing a 37% decrease compared to healthy plants. However, WMV-infected plants and treated with PGPB resulted in increased carbohydrate content. Specifically, soil inoculation with different bacterial strains showed higher carbohydrate contents compared to infected plants. Additionally, seed soaking with *Pseudomonas indica* (OR244617), *Bacillus paramycoides* (OR260281), *Bacillus mycoides* (OR282461), and *Niallia circulans* (OR343128) led to increases of 77%, 52%, 140%, and 75% in carbohydrate content, respectively. A significant increase in carbohydrate content was also observed with foliar inoculation of *Bacillus mycoides* (OR282461) and *Niallia circulans* (OR343128) compared to infected plants (Fig. 6).

### Plant biomass

**Fig. 7:**
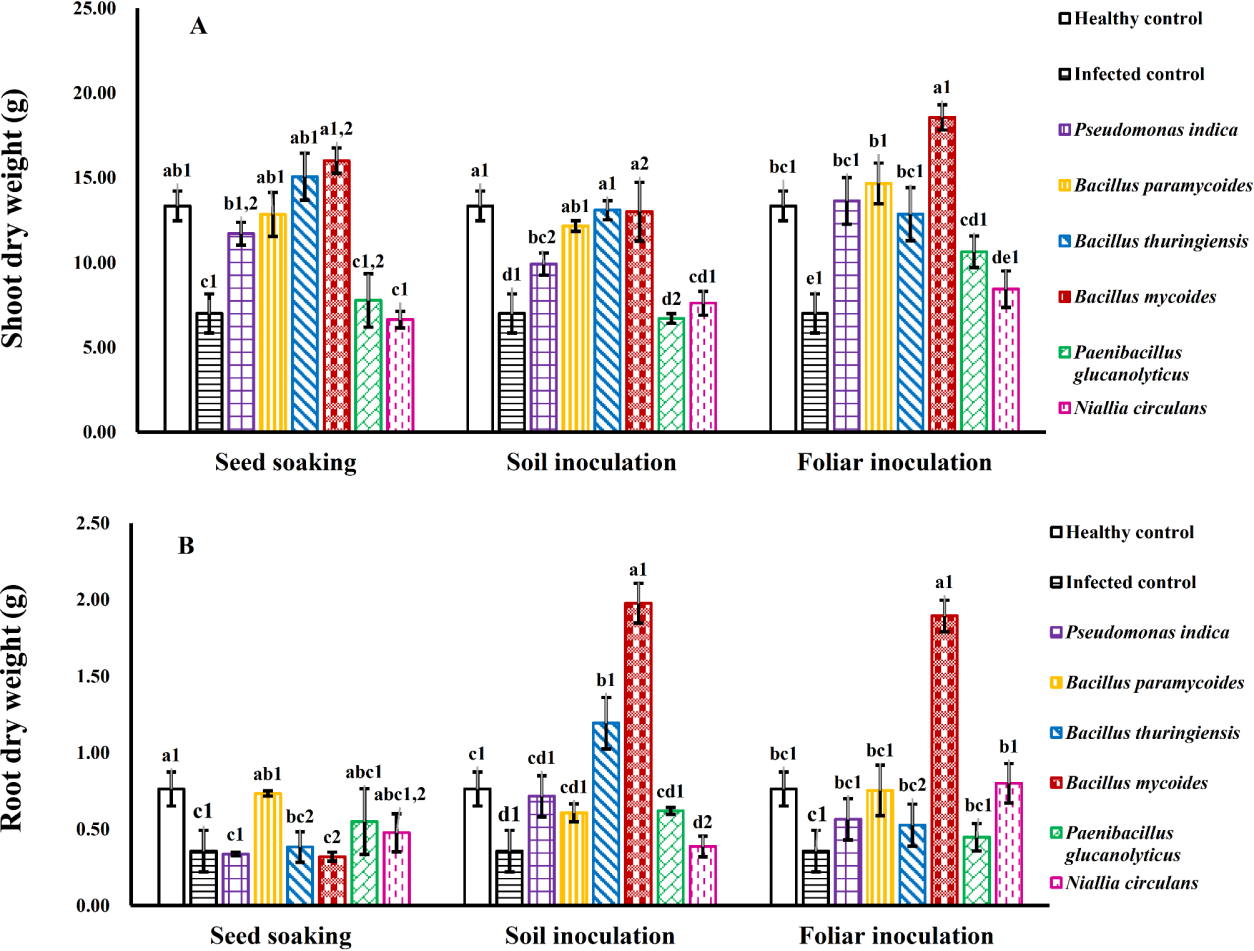
Influence of various bacterial strains and treatment methods on the dry weight of shoots (**A**) and roots (**B**) of squash plants infected with WMV. Each column represents the mean value of three replicates ± SE (vertical bars). Means with the same letters among bacterial isolates and with the same numbers among treatment methods are not significantly different at the 0.05 level using Duncan’s LSD test.

Treatment of WMV-infected squash plants with different strains of PGPB (*P. indica*, *B. paramycoides*, *B. thuringiensis*, and *B. mycoides*) resulted in increased dry weight of shoots compared to infected. However, *Paenibacillus glucanolyticus* and *Niallia circulans* did not show significant increases compared to infected individuals (Fig. 7A).

For root dry weight, no significant differences were observed in WMV-infected squash plants inoculated with *P. indica*, *B. paramycoides*, *Paenibacillus glucanolyticus*, and *Niallia circulans*. However, there was a noticeable difference in plants inoculated with *B. mycoides* using both soil and foliar methods, and *B. thuringiensis* using the soil method (Fig. 7B).

### Disease severity index

**Fig. 8:**
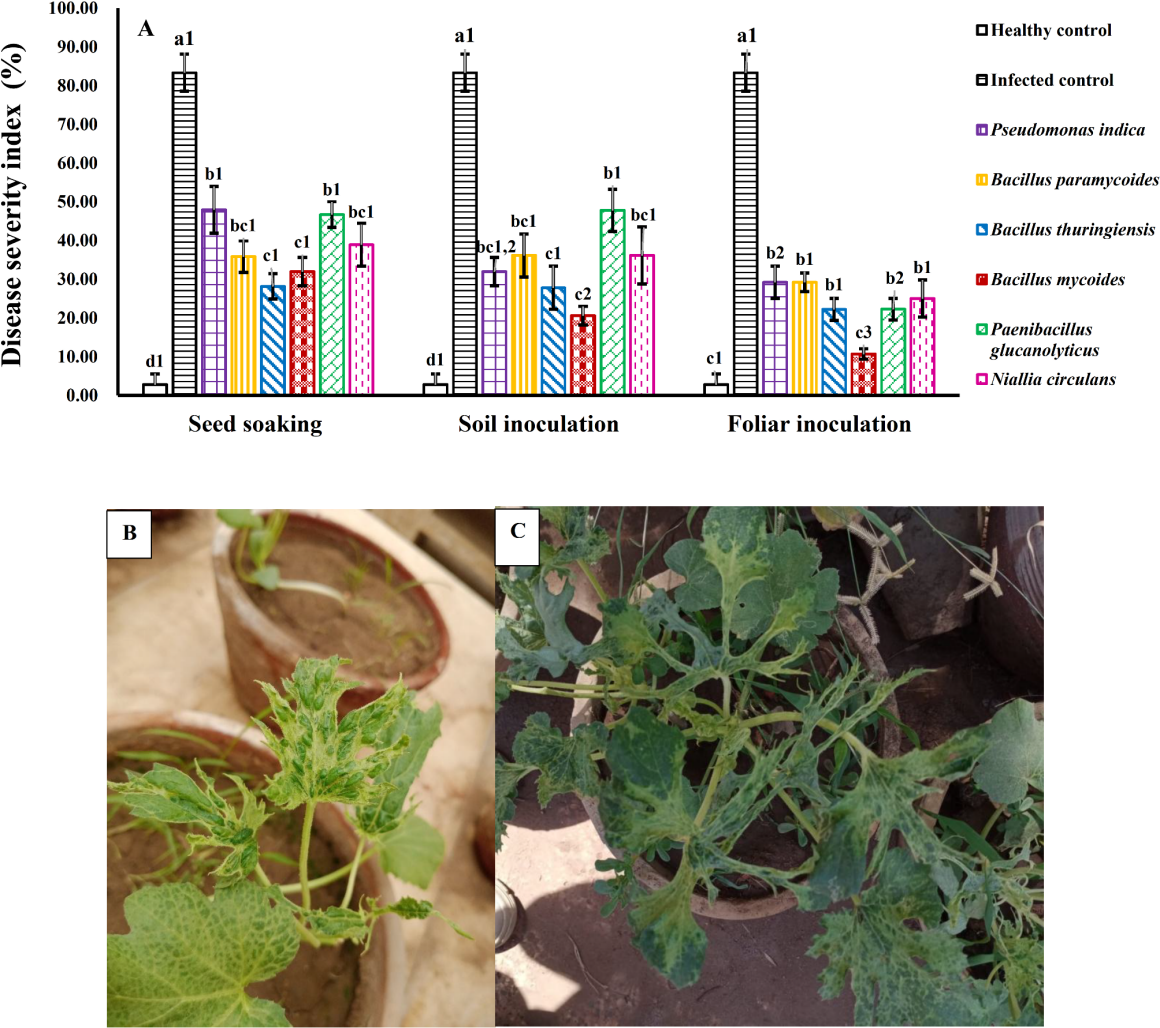
Influence of different bacterial strains and treatment methods on disease severity in squash plants infected with WMV (**A**). Each column represents the mean value of three replicates ± SE (vertical bars). Means with the same letters among bacterial isolates and with the same numbers among treatment methods are not significantly different at the 0.05 level using Duncan LSD test. (**B**,** C**) Photographs showing WMV spread to most of the leaves of the plants (83%) when no treatment was applied.

The disease severity index was determined by assessing the percentage of systemic infection in the leaves, indicating the virus’s ability to spread within the host plant. The results showed that untreated plants had a high level of virus spread, with 83% of leaves infected (Fig. 8). However, plants treated with different PGPB strains showed a significant reduction in systemic infection, ranging from 10.6 to 47%, depending on the strain and inoculation method. Notable differences in the percentage of infected leaves were observed among the strains, with foliar inoculation using *B. mycoides* demonstrating significant variations compared to seed soaking and soil inoculation. These results suggest that foliar inoculation with *B. mycoides* is more effective in reducing systemic WMV infection than seed soaking.

### Gas chromatography–mass spectrometry (GC–MS) analysis

The GC-MS analysis of chemical metabolites produced by six bacterial strains is presented in Table 3. The results show a diverse array of compounds, including fatty acids, esters, and cyclic compounds, highlighting the metabolic capabilities of each strain. Specifically, *Pseudomonas indica* produces 15 metabolites (Supplementary Table S1), *Bacillus paramycoides* generates 18 metabolites (Supplementary Table S2), *Bacillus thuringiensis* contributes 17 metabolites (Supplementary Table S3), *Bacillus mycoides* produces 23 metabolites (Supplementary Table S4), *Paenibacillus glucanolyticus* yields 14 metabolites (Supplementary Table S5), and *Niallia circulans* produces 8 metabolites (Supplementary Table S6). In total, the six bacterial strains produce 73 distinct chemical metabolites. A significant finding is the presence of common metabolites, such as 9-Octadecenoic acid (Z) and their derivatives are prominent across multiple bacterial strains. This suggests a shared metabolic capability among these bacteria, potentially indicating a conserved biosynthetic pathway. The bacterial strains *Bacillus paramycoides*, *Bacillus thuringiensis*, and *Bacillus mycoides* could produce benzene derivatives, such as 1,2-benzenedicarboxylic acid and 1,3-benzenedicarboxylic acid, indicating their potential roles in ecological interactions and plant defense mechanisms. Cyclopentanones are cyclic ketones produced by bacterial strains like *Pseudomonas indica* and *Bacillus paramycoides*. These compounds are present in various natural products and are known for their diverse biological activities. *Niallia circulans* produces compounds such as 9-octadecenoic acid (Z) and pyrrolizin-1,7-dione-6-carboxylic acid, methyl ester, which may enhance plant defenses and exhibit antiviral properties against viruses (Table 3).

**Table 3:** Chemical metabolites identified in Ethyl acetate extracts of *Pseudomonas indica* (*Ps. ind*), *Bacillus paramycoides* (*B. par*), *Bacillus thuringiensis* (*B. thu*), *Bacillus mycoides *(*B. myc*), *Paenibacillus glucanolyticus* (*Pa. glu*), and *Niallia circulans* (*Ni. cir*) using GC–MS analysis.

Chemical compounds		Bacterial strains					
		*Ps. ind*	*B. par*	*B. thur*	*B. myc*	*Pa. gluc*	*Ni cir*
1	9-Octadecenoic acid (Z)-	+	+	+	+	+	+
2	9-Octadecenoic acid (Z)-, methyl ester	-	-	+	+	-	-
3	9-Octadecenoic acid (Z)-, 2-hydroxy-3-[(1-oxohexadecyl)oxy]propyl ester	+	-	-	-	-	-
4	9-Octadecenoic acid (Z)-, phenylmethyl ester	-	-	+	-	-	-
5	9-Octadecenoic acid (Z)-, 2,3-dihydroxypropyl ester	+	-	-	-	-	-
6	9-Octadecenoic acid, 2-(octadecyloxy)ethyl ester	+	-	-	-	-	-
7	9,12-Octadecadienoyl chloride, (Z, Z)-	-	-	+	+	-	-
8	9,12-Octadecadienoic acid (Z, Z)-, methyl ester	-	-	-	+	-	-
9	12,15-Octadecadiynoic acid, methyl ester	-	-	-	+	-	-
10	9,12,15-octadecatrienoic acid	-	-	+	-	+	+
11	Octadecanoic acid, 3-[(1oxohexadecyl)oxy]-2-[(1-oxotetradecyl)oxy]propyl ester	+	-	-	-	-	-
12	Octadecanoic acid, 9,10-epoxy-18-(trimethylsiloxy)-, methyl ester, cis-	-	-	+	-	-	-
13	Octadecanoic acid, 2-oxo-, methyl ester	-	+	-	-	-	-
14	Octadecanal, 2-bromo-	+	-	-	-	+	+
15	Octadecane, 1-iodo-	+	-	-	-	-	-
16	Octadecane, 3-ethyl-5-(2-ethylbutyl)-	-	+	-	-	-	-
17	10-Octadecenal	-	-	-	-	+	-
18	17-Octadecynoic acid	-	+	-	-	-	-
19	12-Methyl-E, E-2,13-octadecadien-1-ol	-	-	-	+	-	-
20	[1,1’-Bicyclopropyl]-2-octanoic acid, 2’-hexyl-, methyl ester	-	-	-	+	-	-
21	[1,1’-Bicyclohexyl]-4-carboxylic acid, 4’-propyl-, 4-fluorophenyl ester	-	+	-	-	-	-
22	1,2 Benzenedicarboxylic acid, butyl 8-methylnonyl ester	+	+	-	-	-	-
23	1,2Benzenedicarboxylic acid, diisooctyl ester	-	+	-	-	-	-
24	1,2-Benzenedicarboxylic acid, mono(2-ethylhexyl) ester	-	+	-	-	-	-
25	1,3-benzenedicarboxylic acid	-	-	-	-	-	+
26	Cyclopentanone, 2-methyl-	+	+	-	-	-	-
27	Cyclopropanepentanoic acid, 2-undecyl-, methyl ester, trans-	-	-	+	+	-	-
28	Tridecanedial	-	-	+	-	-	-
29	2-Bromotetradecanoic acid	-	-	-	+	-	-
30	2-Bromotetradecane	-	+	-	-	-	-
31	7-Methyl-Z-tetradecen-1-ol acetate	-	-	-	-	+	-
32	Tetradecanoic acid, 12-methyl-, methyl ester	-	-	+	-	-	-
33	Pentadecanoic acid,14-methyl-, methyl ester	-	-	+	+	-	-
34	hexadecanoic acid	-	-	+	-	-	-
35	Hexadecanoic acid, 2-[(1 oxotetradecyl)oxy]-1,3-propanediyl ester	+	-	-	-	-	-
36	Hexadecanoic acid, 2-hydroxy-1,3-propanediyl ester	+	-	-	-	-	-
37	Hexadecadienoic acid, methyl ester	-	-	-	+	-	-
38	hexadecanoic acid, 2,3-dihydroxypropyl ester	-	-	-	-	+	-
39	7,11-Hexadecadienal	-	-	-	+	-	-
40	1-Heptatriacotanol	-	-	-	+	+	-
41	9,10 dideutero octadecanal	-	-	-	-	+	+
42	Undecane, 2,4-dimethyl-	+	-	-	-	-	-
43	Dodecane, 2,6,10-trimethyl-	+	-	-	-	-	-
44	Dodecane, 2,6,11-trimethyl-	-	+	-	-	-	-
45	2-Dodecen-1-yl(-)succinic anhydride	-	+	-	-	-	-
46	Dodecane,1-cyclopentyl-4-(3-cyclopentylpropyl)-	-	+	-	-	-	-
47	Eicosapentaenoic Acid, TMS derivative	-	-	-	+	-	-
48	5,8,11,14-Eicosatetraenoic acid, methyl ester, (all-Z)-	-	-	-	+	-	-
49	8,11,14-Eicosatrienoic acid, (Z, Z,Z	-	-	+	-	-	-
50	Ergosta-5,22-dien-3-ol, acetate, (3á,22E)-	-	-	+	-	+	+
51	Cholesta-8,24-dien-3-ol, 4-methyl-, (3á,4à)-	-	-	-	+	-	-
52	Cholestan-3-ol, 2-methylene-, (3á,5à)-	-	-	+	+	-	-
53	cholest-5-en-3-yl palmitate	-	-	-	-	+	-
54	6,9,12,15-Docosatetraenoic acid, methyl ester	-	-	-	+	-	-
55	10,12-Tricosadiynoic acid, methyl ester	-	-	-	+	-	-
56	Oxiraneoctanoic acid, 3-octyl-, cis-	-	-	-	+	-	-
57	Oxalic acid, propyl tridecyl ester	+	-	-	-	-	-
58	Oxalic acid, cyclohexyl tetradecyl ester	-	+	-	-	-	-
59	Isochiapin b	-	-	-	+	+	-
60	Sulfurous acid, hexyl pentadecyl ester	+	+	-	-	-	-
61	Palmitic Acid, TMS derivative	-	-	-	+	-	-
62	Digitoxin	-	-	-	+	-	-
63	Akuammilan-17-ol, 10-methoxy-	-	-	-	-	-	+
64	Pyrrolizin-1,7-dione-6-carboxylic acid, methyl(ester)	-	-	-	-	-	+
65	L-Proline, N-valeryl-, hexadecyl ester	-	+	-	-	-	-
66	1,7-Dimethyl-4-(1-methylethyl)cyclode	-	+	-	-	-	-
67	Eicosane, 9-cyclohexyl-	-	+	-	-	-	-
68	6-epi-shyobunol	-	-	+	-	-	-
69	Trilinolein	-	-	+	-	-	-
70	1,25-Dihydroxyvitamin D3, TMS derivative	-	-	+	-	-	-
71	Cucurbitacin b, 25-desacetoxy-	-	-	-	-	+	-
72	stigmast-5-en-3-ol, (3á,24s)-	-	-	-	-	+	-
73	arabinitol, pentaacetate	-	-	-	-	+	-

## Discussion

### Growth promotion

This study provides the first comprehensive evaluation of six PGPB strains for their ability to promote plant growth and mitigate the impact of WMV on squash plants. The use of six PGPB strains, including *Pseudomonas indica*,* Bacillus paramycoides*,* Bacillus thuringiensis*,* Bacillus mycoides*,* Paenibacillus glucanolyticus*, and *Niallia circulans* as a biocontrol agent to alleviate symptoms of WMV in squash plants and promote plant growth has not been previously documented. *Pseudomonas indica* was initially identified as a PGPB for soybean plants^46^, while *Bacillus paramycoides* has been recognized for its ability to reduce wilt disease severity caused by *F. oxysporum*^47^. *Bacillus thuringiensis* has been studied for its efficacy as a biological control agent against *Salmonella* spp. in fresh produce^48^, and plant pests^49^. *Bacillus mycoides* has shown promise in reducing *Cercospora* leaf spot in sugar beet^50^, and *Paenibacillus lentimorbus* has been identified as a biocontrol agent against cucumber mosaic virus^51^. *Niallia circulans* has been reported to produce IAA and exhibit pest-killing properties^52,53^, with studies showing its ability to enhance plant growth and mitigate root rot stress in eggplant^54^. This study is the first to highlight the potential of these strains as biocontrol agents against WMV in squash plants.

### Biocontrol mechanisms

Previous research has demonstrated that viral infections can stimulate ethylene production in plants, with evidence indicating that ethylene signaling influenced by viruses significantly impacts the susceptibility of host plants^55,56^. For instance, pepper plants infected with broad bean wilt virus 2 (BBWV2) showed increased ethylene production, correlating with more severe disease symptoms^56,57^. Similarly, rice infected with rice dwarf virus exhibited a notable rise in ethylene levels, rendering the plants more susceptible to the virus^58^. Excessive ethylene production can lead to “stress ethylene,” detrimental to root and shoot development and overall plant growth. However, beneficial bacteria containing the enzyme ACCD can mitigate the negative effects of stress ethylene. ACCD breaks down ACC, a precursor to ethylene, into α-ketobutyrate and ammonia, reducing ethylene levels and promoting root and shoot growth^39,59^. In this study, *B. mycoides* (OR282461), *Paenibacillus glucanolyticus* (OR282529), and *N. circulans* (OR343128) exhibited high ACCD activity, suggesting their potential to enhance plant growth and stress tolerance. *Pseudomonas indica* (OR244617) and *Bacillus mycoides* (OR282461) showed moderate ACCD activity, indicating their potential benefits for plant growth under various conditions. Although *B. thuringiensis* (OR272521) lacked ACC deaminase activity, it possesses other attributes that could contribute to improving plant growth.

### Chemical metabolites

GC-MS analysis of chemical metabolites from six bacterial strains reveals a diverse range of compounds, including fatty acids, esters, and cyclic compounds, showcasing the unique metabolic capabilities of each strain. This metabolic diversity is crucial for combating WMV and promoting overall plant health. The six bacterial strains collectively produce 73 distinct chemical metabolites, highlighting their potential to bolster plant defenses. The presence of shared metabolites, like 9-octadecenoic acid (Z) and its derivatives across multiple strains, indicates a conserved metabolic pathway. This shared capability could be harnessed to develop consistent strategies for plant protection. Fatty acids and esters produced by *Pseudomonas indica* and *Paenibacillus glucanolyticus* play crucial roles in plant defense mechanisms. These compounds strengthen plant cell walls, making it harder for pathogens like WMV to invade and infect plant tissues. Additionally, fatty acids can act as signaling molecules that trigger systemic acquired resistance, enhancing the plant’s overall immunity^60^. 9-octadecenoic acid (Z), which are involved in altering salicylic acid and jasmonic acid mediated defense responses, enhancing plant immunity against pathogens, including viruses^61^. The synthesis of benzene derivatives by *Bacillus paramycoides*,* Bacillus thuringiensis* and *Bacillus mycoides* is noteworthy. Compounds like 1,2-benzenedicarboxylic acid and 1,3-benzenedicarboxylic acid have ecological roles and contribute to plant defense. These derivatives may serve as antimicrobial agents^62–64^, inhibiting the growth of pathogenic fungi and bacteria, thereby reducing secondary infections in plants already stressed by WMV. 9-octadecenoic acid (Z)-methyl ester and Pentadecanoic acid, 14-methyl-, methyl ester have antifungal properties, while 9-octadecenoic acid (Z)-, phenylmethyl ester has antimicrobial properties^65^. Cyclopentanones produced by *Pseudomonas indica* and *Bacillus paramycoides* are cyclic ketones with diverse biological activities, including antimicrobial properties^66,67^. Cyclopentanones have been studied for their broad-spectrum antiviral properties, including their potential to inhibit plant viruses. These compounds can interfere with viral replication and enhance plant immune responses^68^. Their presence in the metabolites suggests a potential role in directly inhibiting WMV or other co-infecting pathogens, providing additional protection to the plant. *Niallia circulans* produces unique metabolites, such as 9-octadecenoic acid (Z) and pyrrolizin-1,7-dione-6-carboxylic acid methyl ester, which may enhance plant defenses and exhibit antiviral properties against WMV. These compounds could interfere with viral replication or strengthen the plant’s innate immune responses^69^. The diverse chemical metabolites produced by the six bacterial strains demonstrate their potential to combat WMV and enhance plant health. Shared metabolites indicate a common biosynthetic pathway that can be utilized for effective plant protection. The unique metabolites from each strain suggest a multifaceted approach to boosting plant defenses, combining different bacterial metabolites to create robust protection against WMV and other pathogens. This study underscores the importance of using microbial metabolites in sustainable agriculture to improve crop resilience and productivity.

### Broader implications

Recently, studies have shown that bioactive compounds from bacterial supernatants can combat plant viruses through mechanisms like systemic resistance induction and defensive enzyme production^70^. Direct inhibition of viral replication and effects on virus vectors have also been observed^16^. Compounds in the bacterial extracts, such as cyclopentanone, 2-methyl, may disrupt viral replication processes^71^, while 9-octadecenoic acid, 2-(octadecyloxy) ethyl ester, with antioxidant properties, can mitigate oxidative stress during viral infections^72^. Palmitic acid, known for protein palmitoylation, can influence protein function in plant defense mechanisms^73,74^ and pentadecanoic acid may enhance the plant’s immune response by modulating defense-related metabolites. In a previous study by^75^, bioactive compounds in the ethyl acetate extract of *Bacillus subtilis* strain HA1 culture filtrate were identified, including eicosane and pentadecanoic acid, suggesting their role in inducing systemic resistance in tomato plants against viral infections. These findings contribute to understanding the potential impacts of bacterial extracts on viral infections in plants and highlight the diverse bioactive properties of the identified compounds. Moreover, Stigmast-5-en-3-ol (beta-sitosterol) has been found to stabilize cell membranes and may enhance the plant’s resistance to viral entry and spread. Also, Stigmast-5-en-3-ol (beta-sitosterol) has been found in plants like *Calotropis procera*, where it contributes to the plant’s overall immunomodulatory activity and could potentially enhance the plant’s resistance to viral entry and spread^76^. Stigmast-5-en-3-ol, a type of phytosterol, is known for its various biological effects, including potential antiviral properties. While specific studies on its direct effects on plant viruses are limited, phytosterols like stigmast-5-en-3-ol may help in stabilizing cellular membranes and enhancing plant defense mechanisms, which could indirectly provide some resistance against viral infections^77,78^. More research is needed to fully elucidate its efficiency in combating specific plant viruses. Furthermore, the sterol Ergosta-5,22-dien-3-ol has been isolated from *T. heimii* and has antimicrobial properties Ergosterol and ergosta-5,8-dien-3-ol hold great promise as new drugs against hepatocarcinoma^79^. Pyrrolizin-1,7-dione-6-carboxylic acid, methyl ester, part of the pyrrolizidine alkaloids, produces by many plant species. It is known for its defensive role in plants, potentially deterring viral pathogens^80^. There is no publication record that Stigmast-5-en-3-ol, Ergosta-5,22-dien-3-ol and Pyrrolizin-1,7-dione-6-carboxylic acid, methyl ester produced by bacteria. These compounds collectively contribute to the plant’s defense system, enhancing its ability to resist and recover from viral infections. In this study, Digitoxin has been identified in the mass analysis of the bacterial metabolite from *Bacillus mycoides*. Some studies have recognized its potential antiviral properties, which can effectively inhibit the replication of human cytomegalovirus by inducing autophagy. This process is facilitated through the activation of AMP-activated protein kinase and the Na^+^/K^+^ ATPase α1 subunit, which disrupts the virus’s replication capabilities within host cells^81^. Furthermore, digitoxin and its analogues have also exhibited antiviral effects against other viruses, such as herpes simplex virus and certain strains of cytomegalovirus^82^. Their specific mechanisms can include disrupting viral replication, stabilizing cell structures, and boosting the plant’s overall immune response. These findings underscore the multifaceted approach of bioactive compounds in managing plant virus infections, making them a valuable tool in sustainable agriculture. In summary, the bioactive compounds in bacterial supernatants offer a comprehensive strategy to manage plant virus infections, combining systemic resistance, direct antiviral activity, and vector control.

Moreover, Stigmast-5-en-3-ol (beta-sitosterol) has been identified as a compound that can stabilize cell membranes, potentially enhancing a plant’s resistance to viral entry and spread. This phytosterol is present in plants like *Calotropis procera*, contributing to their immunomodulatory activity and possibly bolstering their defense against viral infections^76^. While research on the direct antiviral effects of Stigmast-5-en-3-ol is limited, its ability to stabilize cellular membranes and boost plant defense mechanisms may indirectly confer resistance to viral infections^77,78^. Ergosta-5,22-dien-3-ol, a sterol isolated from *T. heimii*, exhibits antimicrobial properties and shows promise as a potential treatment for hepatocarcinoma^79^. Pyrrolizin-1,7-dione-6-carboxylic acid, methyl ester, a pyrrolizidine alkaloid found in various plant species, plays a defensive role in plants and may act as a deterrent against viral pathogens^80^. While these compounds are not known to be produced by bacteria, they collectively contribute to a plant’s defense system, aiding in resistance and recovery from viral infections. Their mechanisms may involve disrupting viral replication, stabilizing cell structures, and enhancing the plant’s immune response. These bioactive compounds demonstrate a multifaceted approach to managing plant virus infections, offering valuable tools for sustainable agriculture.

The findings of this study highlight the potential of Plant Growth-Promoting Bacteria (PGPB) as a sustainable alternative to chemical pesticides in managing WMV in squash. Specific bacterial strains, particularly *Bacillus mycoides*, have been shown to significantly reduce the severity of viral infections and enhance plant growth, offering a promising strategy for sustainable agriculture. Integrating PGPB into agricultural practices can serve as a biological control method, reducing the need for chemical interventions and minimizing environmental contamination. PGPB can improve soil health, nutrient availability, and plant growth, leading to more resilient crops capable of withstanding various stresses. By promoting crop rotation and diversity, PGPB can enhance ecosystem resilience and reduce the prevalence of crop-specific pests and diseases^83,84^. Utilizing natural biocontrol agents allows for targeted management of viral infections without the broad-spectrum impact of chemical pesticides, ultimately lowering overall pesticide use and production costs for farmers^85,86^. This shift towards sustainable practices can improve food safety, public health, and crop yields, contributing to global food security challenges exacerbated by climate change. By aligning with international goals for resilient food systems, the study emphasizes the importance of reducing chemical dependency in agriculture and promoting sustainable methods like PGPB integration.

### Future prospect

To advance research on PGPB and WMV, several concrete next steps can be undertaken. First, conducting experiments to evaluate the effectiveness of PGPB across diverse environmental conditions, such as varying soil types, climates, and water availability, will help determine the robustness and adaptability of these bacterial strains in practical agricultural settings. Second, developing formulations or delivery systems to enhance the stability and efficacy of PGPB is essential. This could involve techniques like encapsulation, slow-release formulations, or combining PGPB with other beneficial soil amendments to optimize their performance in the field. Finally, exploring the potential synergistic effects between different bacterial strains or their metabolites through co-culturing can reveal whether combined applications yield greater plant growth and disease resistance compared to individual strains. By focusing on these aspects, researchers can significantly enhance the practical application of PGPB in managing WMV, maximizing their benefits under various conditions.

## Conclusion

This research effectively identified WMV in squash plants through RT-PCR and symptom analysis. Additionally, it demonstrated the beneficial effects of various bacterial treatments on plants infected with WMV. The improvements in plant height, pigment content, carbohydrate levels, and overall growth highlight the potential of PGPB in enhancing the health of infected squash. *Bacillus mycoides* treatment proved to be the most effective PGPB, reducing the severity of WMV) in squash plants by 87%. Additionally, it increased carbohydrate content by 140% and enhanced chlorophyll and carotenoid levels by 77%. The treatment also led to a significant increase in plant height. The bioactive compounds in bacterial supernatants present a comprehensive approach to managing viral infections by promoting systemic resistance, exhibiting direct antiviral activity, and aiding in vector control. GC-MS analysis identified 73 chemical metabolites from six bacterial strains, some of which act as elicitor molecules to boost plant defenses against WMV. The discovery of common metabolites, such as 9-Octadecenoic acid (Z), suggests shared metabolic pathways among strains, facilitating the development of consistent and effective plant protection strategies. This integrated approach not only offers a promising solution for controlling viral infections in crops but also contributes significantly to enhancing agricultural sustainability and efficiency. Future research could validate these findings through field trials to assess the efficacy of bacterial treatments under real-world conditions. Exploring the impact of these treatments on other crops infected with WMV or related viruses could provide broader insights into their potential applications. Further studies could investigate the mechanisms by which these bacterial treatments enhance plant resistance or reduce viral load, as well as their long-term effects on plant health and yield.

## Electronic supplementary material

Below is the link to the electronic supplementary material.

Supplementary Material 1

## Data Availability

The nucleotide sequence of bacterial isolates (OA2, OA7, OA8, OA15, OA52, OA56) isolated from basil (*Ocimum basilicum*), mint (*Mentha viridis*), thyme (*Thymus vulgaris*), and squash (*Cucurbita pepo* L) plants were deposited in the GenBank nucleotide sequence database under accession number.

## Abbreviations

PGPB: Plant growth-promoting bacteria
WMV: Watermelon mosaic virus
ACCD: 1-aminocyclopropane-1-carboxylate deaminase
