# Supplementary material for: Harnessing plant growth-promoting bacteria to combat watermelon mosaic virus in squash
Source: Sci Rep. 2025 Mar 19;15:9440. doi: 10.1038/s41598-025-92268-2 (PMC11923214; doi:10.1038/s41598-025-92268-2)
Supplement: Supplementary file 1 — Supplementary Material 1 [file 41598_2025_92268_MOESM1_ESM.docx]

**Harnessing plant growth-promoting bacteria to combat watermelon mosaic virus in squash**

Shymaa R.Bashandy^1^, Omima Abdelsater Mohamed^1^, O.A. Abdalla^2^, A Elfarash^3,^

Mohamed Hemida Abd-Alla^1*^

^1^Botany and Microbiology Department, Faculty of Science, Assiut University, Assiut 71516, Egypt

^2^ Plant Pathology Department Faculty of Agriculture, Assiut University

^3^Genetics Department Faculty of Agriculture, Assiut University

*Corresponding author: Prof Dr Mohamed Hemida Abd-Alla,

E mail: [mhabdalla@aun.edu.eg](mailto:mhabdalla@aun.edu.eg); mhabdalla2002@yahoo.com

**ORCID ID: 0000-0003-0415-9409**

**Running title**: **Beneficial Bacteria Combat WMV in Squash**.


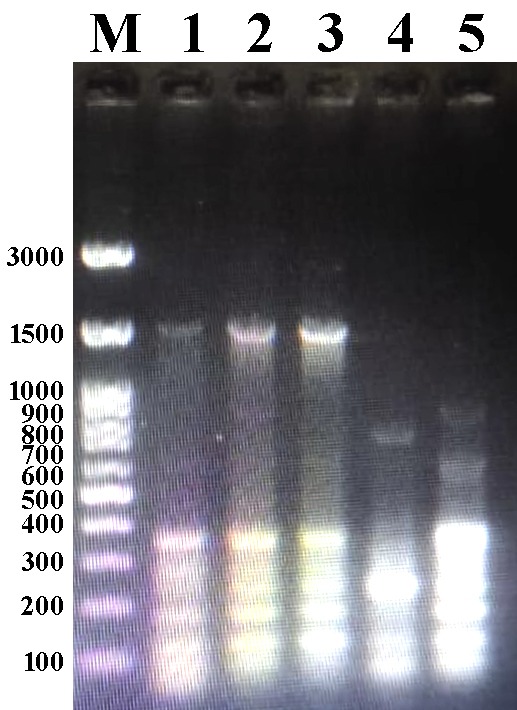


**Supplementary Figure S1**

**Supplementary Table** **S1 showing the chemical properties of the compounds of ethyl acetate extract of *Pseudomonas indica* (*Ps. ind*) isolate filtrate using GC–MS analysis**.

| Peak NO | Retention Time (RT) | Compound Name | Area | Molecular formula | Molecular weight |
| --- | --- | --- | --- | --- | --- |
| 1 | 4.21 | 9-Octadecenoic acid, 2-(octadecyloxy)ethyl ester | 0.31 | C38H74O3 | 578 |
| 2 | 4.40 | 9-Octadecenoic acid (Z)-, 2,3-dihydroxypropyl ester | 0.30 | C21H40O4 | 356 |
| 3 | 5.44 | Hexadecanoic acid, 2-[(1 oxotetradecyl)oxy]-1,3-propaned iyl ester | 0.17 | C49H94O6 | 778 |
| 4 | 6.75 | Octadecanoic acid, 3-[(1oxohexadecyl)oxy]-2-[(1-oxotetra decyl)oxy]propyl ester | 0.29 | C51H98O6 | 806 |
| 5 | 7.72 | Hexadecanoic acid, 2-hydroxy-1,3 propanediyl ester | 0.19 | C35H68O5 | 568 |
| 6 | 7.79 | 9-Octadecenoic acid (Z)-, 2-hydroxy-3-[(1 oxohexadecyl)oxy]pro pyl ester | 0.23 | C37H70O5 | 594 |
| 7 | 8.09 | Cyclopentanone, 2-methyl- | 23.55 | C6H10O | 98 |
| 8 | 45.56 | Undecane, 2,4-dimethyl- | 0.17 | C13H28 | 184 |
| 9 | 51.12 | Octadecane, 1-iodo- | 0.22 | C18H37I | 380 |
| 10 | 57.22 | Oxalic acid, propyl tridecyl ester | 0.18 | C18H34O4 | 314 |
| 11 | 58.37 | 1,2 Benzenedicarboxylic acid, butyl 8-methylnonyl ester | 0.28 | C22H34O4 | 362 |
| 12 | 59.09 | Dodecane, 2,6,10-trimethyl- | 0.63 | C15H32 | 212 |
| 13 | 62.07 | Sulfurous acid, hexyl pentadecyl ester | 0.35 | C21H44O3S | 376 |
| 14 | 83.72 | 9-octadecenoic acid (z)- | 0.18 | C18H34O2 | 282 |
| 15 | 84.05 | Octadecanal, 2-bromo- | 0.17 | C18H35BrO | 346 |

**Supplementary Table S2 showing the chemical properties of the compounds of ethyl acetate extract of *Bacillus paramycoides* (*B. par*) isolate filtrate using GC–MS analysis.**

| Peak NO | Retention  Time (RT) | Compound Name | Area | Molecular  formula | Molecular weight |
| --- | --- | --- | --- | --- | --- |
| 1 | 8.02 | Cyclopentanone, 2-methyl- | 22.91 | C6H10O | 98 |
| 2 | 51.06 | Dodecane, 2,6,11-trimethyl- | 0.36 | C15H32 | 212 |
| 3 | 55.04 | Oxalic acid, cyclohexyl tetradecyl ester | 0.18 | C22H40O4 | 368 |
| 4 | 55.65 | 2-Bromotetradecane | 0.52 | C14H29Br | 276 |
| 5 | 79.13 | 1,2Benzenedicarboxylic acid, diisooctyl ester | 67.46 | C24H38O4 | 390 |
| 6 | 81.56 | 2-Dodecen-1-yl(-)succinic anhydride | 0.13 | C16H26O3 | 266 |
| 7 | 57.16 | Octadecane, 3-ethyl-5-(2-ethylbutyl)- | 0.16 | C26H54 | 366 |
| 8 | 57.78 | L-Proline, N-valeryl-, hexadecyl ester | 0.24 | C26H49NO3 | 423 |
| 9 | 58.33 | 1,2-Benzenedicarboxylic acid, butyl 8-methylnonyl ester | 0.26 | C22H34O4 | 362 |
| 10 | 58.88 | 1,7-Dimethyl-4-(1 methylethyl)cyclodecane | 0.23 | C15H30 | 210 |
| 11 | 59.06 | Sulfurous acid, hexyl pentadecyl ester | 0.86 | C21H44O3S | 376 |
| 12 | 59.81 | Dodecane,1-cyclopentyl-4-(3-cyclopentylpropyl)- | 0.14 | C25H48 | 348 |
| 13 | 66.95 | Eicosane, 9-cyclohexyl- | 0.17 | C26H52 | 364 |
| 14 | 77.40 | 9-octadecenoic acid (z)- | 0.20 | C18H34O2 | 282 |
| 15 | 69.76 | Octadecanoic acid, 2-oxo-, methyl ester | 0.13 | C19H36O3 | 312 |
| 16 | 79.13 | 1,2-Benzenedicarboxylic acid, mono(2-ethylhexyl) ester | 67.46 | C16H22O4 | 278 |
| 17 | 84.34 | 17-Octadecynoic acid | 0.19 | C18H32O2 | 280 |
| 18 | 88.50 | [1,1'-Bicyclohexyl]-4-carboxylic acid, 4'-propyl-, 4-fluorophenyl ester | 0.17 | C22H31FO2 | 346 |

**Supplementary** **Table S3 showing the chemical properties of the compounds of ethyl acetate extract of *Bacillus thuringiensis* (*B. thu*) isolate filtrate using GC–MS analysis.**

| Peak No | Retention Time (RT) | Compound Name | Area | Molecular formula | Molecular weight |
| --- | --- | --- | --- | --- | --- |
| 1 | 14.18 | 9-Octadecenoic acid (Z)-, phenylmethyl ester | 0.45 | C25H40O2 | 372 |
| 2 | 23.20 | Cyclopropanepentanoic acid, 2-undecyl-, methyl ester, trans- | 0.86 | C20H38O2 | 310 |
| 3 | 27.32 | Pentadecanoic acid, 14-methyl-, methyl ester | 11.05 | C17H34O2 | 270 |
| 4 | 28.29 | hexadecanoic acid | 12.53 | C16H32O2 | 256 |
| 5 | 30.59 | 9-Octadecenoic acid (Z)-, methyl ester | 9.33 | C19H36O2 | 296 |
| 6 | 30.72 | 9,12-Octadecadienoyl chloride, (Z,Z)- | 2.33 | C18H31ClO | 298 |
| 7 | 31.14 | Tetradecanoic acid, 12-methyl-, methyl ester | 3.33 | C16H32O2 | 256 |
| 8 | 31.93 | 9-octadecenoic acid (z)- | 0.53 | C18H34O2 | 282 |
| 19 | 32.10 | 8,11,14-Eicosatrienoic acid, (Z,Z,Z | 1.89 | C20H34O2 | 306 |
| 10 | 33.08 | Cholestan-3-ol, 2-methylene-, (3á,5à) | 1.24 | C28H48O | 400 |
| 11 | 35.91 | Tridecanedial | 0.90 | C13H24O2 | 212 |
| 12 | 36.27 | 6-epi-shyobunol | 1.12 | C15H26O | 222 |
| 13 | 37.42 | 9,12,15-octadecatrienoic acid | 0.48 | C25H40O6 | 436 |
| 14 | 37.89 | Trilinolein | 1.49 | C57H98O6 |  |
| 15 | 39.10 | 1,25-Dihydroxyvitamin D3, TMS derivative | 0.36 | C30H52O3Si | 488 |
| 16 | 40.27 | octadecanoic acid, 9,10-epoxy-18-(trimethylsiloxy)-, methyl ester, cis- | 0.93 | C22H44O4Si | 400 |
| 17 | 40.34 | Ergosta-5,22-dien-3-ol, acetate, (3á,22E)- | 0.78 | C30H48O2 | 440 |

**Supplementary Table Table S 4 showing the chemical properties of the compounds of ethyl acetate extract of *Bacillus mycoides* (*B. myc*) isolate filtrate using GC–MS analysis.**

| Peak No | Retention  Time (RT) | Compound Name | Area | Molecular formula | Molecular weight |
| --- | --- | --- | --- | --- | --- |
| 1 | 33.02 | 5,8,11,14-Eicosatetraenoic acid, methyl ester, (all-Z)- | 0.77 | C21H34O2 | 318 |
| 2 | 36.07 | pentadecanoic acid,14-methyl-, methyl ester | 8.74 | C17H34O2 | 270 |
| 3 | 37.11 | 12,15-Octadecadiynoic acid, methyl ester | 0.73 | C19H30O2 | 290 |
| 4 | 39.79 | Palmitic Acid, TMS derivative | 17.83 | C19H40O2Si | 328 |
| 5 | 40.44 | 9,12-octadecadienoic acid (z,z)-, methyl ester | 1.70 | C19H34O2 | 294 |
| 6 | 40.67 | 9-Octadecenoic acid (Z)-, methyl ester | 6.22 | C19H36O2 | 296 |
| 7 | 40.86 | hexadecadienoic acid, methyl ester | 1.51 | C17H30O2 | 266 |
| 8 | 41.51 | Cyclopropanepentanoic acid, 2-undecyl-, methyl ester, trans- | 1.87 | C20H38O2 | 310 |
| 9 | 41.99 | 7,11-Hexadecadienal | 5.40 | C16H28O | 236 |
| 10 | 42.08 | 9-octadecenoic acid (z)- | 3.84 | C18H34O2 | 282 |
| 11 | 44.22 | 9,12-Octadecadienoyl chloride, (Z,Z)- | 1.81 | C18H31ClO | 298 |
| 12 | 44.55 | [1,1'-Bicyclopropyl]-2-octanoic acid, 2'-hexyl-, methyl ester | 5.22 | C21H38O2 | 322 |
| 13 | 45.78 | Eicosapentaenoic Acid, TMS  derivative | 0.78 | C23H38O2Si | 374 |
| 14 | 53.81 | 6,9,12,15-Docosatetraenoic acid, methyl ester | 0.74 | C23H38O2 | 346 |
| 15 | 53.90 | 5,8,11,14-Eicosatetraenoic acid, methyl ester, (all-Z)- | 0.47 | C21H34O2 | 318 |
| 16 | 55.42 | Oxiraneoctanoic acid, 3-octyl-, cis- | 2.18 | C18H34O3 | 298 |
| 17 | 59.39 | isochiapin b | 0.82 | C19H22O6 | 346 |
| 18 | 60.73 | 1-Heptatriacotanol | 0.83 | C37H76O | 536 |
| 19 | 60.82 | 10,12-Tricosadiynoic acid, methyl ester | 0.79 | C24H40O2 | 360 |
| 20 | 63.77 | Cholesta-8,24-dien-3-ol, 4-methyl-, (3á,4à)- | 0.35 | C28H46O | 398 |
| 21 | 68.38 | 12-Methyl-E,E-2,13-octadecadien-1- ol | 0.44 | C19H36O | 280 |
| 22 | 69.50 | Cholestan-3-ol, 2-methylene-,(3á,5à)- | 3.73 | C28H48O | 400 |
| 23 | 78.22 | 2-Bromotetradecanoic acid | 0.43 | C14H27BrO2 | 306 |
| 24 | 80.52 | Digitoxin | 0.48 | C41H64O13 | 764 |

**Supplementary Table S5 showing the chemical properties of the compounds of ethyl acetate extract of *Paenibacillus glucanolyticus* (*Pa. glu*) isolate filtrate using GC–MS analysis.**

| Peak No | Retention  Time (RT) | Compound Name | Area | Molecular formula | Molecular weight |
| --- | --- | --- | --- | --- | --- |
| 1 | 16.53 | 9-octadecenoic acid (z)- | 0.26 | C18H34O2 | 282 |
| 2 | 18.99 | 7-Methyl-Z-tetradecen-1-ol acetate | 0.35 | C17H32O2 | 268 |
| 3 | 24.98 | 9,12,15-octadecatrienoic acid | 0.25 | C28H40O4 | 440 |
| 4 | 26.90 | hexadecanoic acid, 2,3-dihydroxypropyl ester | 0.23 | C19H38O4 | 330 |
| 5 | 29.97 | Octadecanal, 2-bromo- | 0.26 | C18H35BrO | 346 |
| 6 | 53.66 | 1-Heptatriacotanol | 0.17 | C37H76O | 536 |
| 7 | 53.85 | isochiapin b | 0.38 | C19H22O6 | 346 |
| 8 | 58.52 | 10-Octadecenal | 0.36 | C18H34O | 266 |
| 9 | 65.43 | 9,10 dideutero octadecanal | 0.28 | C18H34D2O | 270 |
| 10 | 66.21 | Ergosta-5,22-dien-3-ol, acetate, (3á,22E)- | 9.54 | C30H48O2 | 440 |
| 11 | 67.85 | Cucurbitacin b, 25-desacetoxy- | 1.88 | C30H44O6 | 500 |
| 12 | 68.58 | cholest-5-en-3-yl palmitate | 0.29 | C43H76O2 | 624 |
| 13 | 70.96 | stigmast-5-en-3-ol, (3á,24s)- | 31.23 | C29H50O | 414 |
| 14 | 73.84 | arabinitol, pentaacetate | 0.33 | C15H22O10 | 362 |

**Supplementary Table S6 showing the chemical properties of the compounds of ethyl acetate extract of *Niallia circulans* (*Ni cir*) isolate filtrate using GC–MS analysis.**

| Peak No | Retention  Time  (RT) | Compound Name | Area | Molecular formula | Molecular weight |
| --- | --- | --- | --- | --- | --- |
| 1 | 5.59 | Akuammilan-17-ol, 10-methoxy- | 6.22 | C20H24N2O2 | 324 |
| 2 | 8.64 | 9,12,15-octadecatrienoic acid | 4.83 | C28H40O4 | 440 |
| 3 | 20.15 | 1,3-benzenedicarboxylic acid | 7.34 | C8H6O4 | 166 |
| 4 | 35.86 | Pyrrolizin-1,7-dione-6-carboxylic acid, methyl(ester) | 12.53 | C9H11NO4 | 186 |
| 5 | 35.94 | Octadecanal, 2-bromo- | 4.70 | C18H35BrO | 346 |
| 6 | 40.54 | 9-octadecenoic acid (z)- | 19.53 | C18H34O2 | 282 |
| 7 | 62.50 | Ergosta-5,22-dien-3-ol, acetate, (3á,22E)- | 6.42 | C30H48O2 | 440 |
| 8 | 67.93 | 9,10 dideutero octadecanal | 4.73 | C18H34D2O | 270 |
